# Impacts of Supplementing Broiler Diets with Biological Curcumin, Zinc Nanoparticles and *Bacillus licheniformis* on Growth, Carcass Traits, Blood Indices, Meat Quality and Cecal Microbial Load

**DOI:** 10.3390/ani11071878

**Published:** 2021-06-24

**Authors:** Mohamed E. Abd El-Hack, Bothaina A. Alaidaroos, Reem M. Farsi, Diaa E. Abou-Kassem, Mohamed T. El-Saadony, Ahmed M. Saad, Manal E. Shafi, Najah M. Albaqami, Ayman E. Taha, Elwy A. Ashour

**Affiliations:** 1 Poultry Department, Faculty of Agriculture, Zagazig University, Zagazig 44511, Egypt; elwynurition@yahoo.com; 2 Department of Biological Sciences, Faculty of Science, King Abdulaziz University, Jeddah 21577, Saudi Arabia; b.alaidaros@kau.edu.sa (B.A.A.); rfarsi@kau.edu.sa (R.M.F.); meshafi@kau.edu.sa (M.E.S.); nalbogami@kau.edu.sa (N.M.A.); 3 Animal and Poultry Production Department, Faculty of Technology and Development, Zagazig University, Zagazig 44519, Egypt; alaa1.aa837@gmail.com; 4 Department of Agricultural Microbiology, Faculty of Agriculture, Zagazig University, Zagazig 44511, Egypt; m_tlatelsado@yahoo.com; 5 Biochemistry Department, Faculty of Agriculture, Zagazig University, Zagazig 44511, Egypt; ahmedm4187@gmail.com; 6 Department of Animal Husbandry and Animal Wealth Development, Faculty of Veterinary Medicine, Alexandria University, Edfina 22756, Egypt; a.taha@alexu.edu.eg

**Keywords:** ZnNPs, CurNPs, *Bacillus*, broilers, growth, microbial aspects

## Abstract

**Simple Summary:**

The present study aimed to investigate the beneficial effects of zinc nanoparticles (ZnNPs) and curcumin nanoparticles (CurNPs) as well as *Bacillus licheniformis* (Bl) supplementation on broiler growth, chemical blood indices, and cecal microbes. The results showed considerable antimicrobial activity against pathogenic bacteria and fungi with ZnNPs and CurNPs supplementations. At the same time, ZnNPs, CurNPs, and Bl improved broiler performance, carcass traits, meat quality traits, and some blood indices. Therefore, the inclusion of ZnNPs, CurNPs, and Bl is recommended for broiler feeding regimens to improve the performance and health status.

**Abstract:**

The current study aimed to investigate the influence of dietary zinc nanoparticles (ZnNPs), curcumin nanoparticles (CurNPs), and *Bacillus licheniformis* (Bl) on the growth, carcass, blood metabolites, and the count of some cecal microorganisms of Indian River (IR) broilers. Chicks were allotted into seven experimental groups: control group, 1st, 2nd and 3rd groups were given diets enriched with ZnNPs, CurNPs and Bl (3.0, 5.0 and 2.0 cm^3^/kg diet, respectively). The 4th, 5th and 6th groups were given diets supplemented with ZnNPs (3.0) + Bl (2.0) (ZP); ZnNPs (3.0) + CurNPs (5.0) (ZC) and ZnNPs (3.0) + CurNPs (5.0) + Bl (2.0) (ZCP) cm^3^/kg diet, respectively. The results revealed that ZnNPs and CurNPs exhibited a considerable antimicrobial activity against pathogenic bacteria and fungi. They also inhibited the growth of microbes in a range of 50–95 µg/mL. The diet supplemented with ZnNPs, CurNPs, and Bl increased the body weight compared to the control after five weeks of age. Additionally, values of daily feed intake increased in these groups; however, the feed conversion ratio decreased. All values of carcass traits were better than that of the control. The treatments led to decreased abdominal lipids compared to the control. The activity of liver enzymes and malondialdehyde (MDA) activity decreased in the treated groups. In a converse trend, the levels of oxidative enzymes, amylase, protease, lipase and immunoglobulin were higher than that of the control. Meat quality properties were improved and cecal microbial counts were decreased. In conclusion, the ZnNPs, CurNPs, and Bl improved the broiler’s weights, carcass traits, meat quality traits, as well as some blood indices and cecal microbial load. Therefore, the inclusion of ZnNPs, CurNPs, or Bl is recommended for broiler feeding regimens to improve the performance and health status.

## 1. Introduction

In the poultry industry, the main goal is to supply safer feed to enhance performance and health [1]. In 2006, the European Union banned the addition of antibiotics to farm animal diets to avoid microbial resistance to antibiotics [2]. The use of natural feed additives in broiler diets such as plants and their derivatives and the study of their effect on the quantity and quality of poultry are now global trends [3,4,5,6,7].

Nanoparticles (NPs) can be synthesized using physical, chemical, and biological methods. The biological synthesis is safe, clean, biocompatible, eco-friendly, and accomplishes fast reduction of metal ions at room temperature, unlike physical or chemical methods that consume extensive energy or use toxic solvents, respectively [8,9,10]. Microbes are potent eco-friendly nano-factories and can control the size and shape of biological nanoparticles [11]. Nanoparticles offer excellent properties, such as a large surface area, increased catalytic activity, and powerful adsorption capacity [12]. There are signs that nanoparticles can raise the absorption of nutrients [13].

Zinc (Zn) is an important microelement that affects many biological processes in birds, i.e., carbohydrates, protein and fat metabolism, immunity, hormone building, DNA and protein synthesis, and antioxidant properties [14]. According to the National Research Council (NRC), poultry needs 40 ppm of zinc per day [15]. On a commercial scale, feed industrialists add an extra amount of zinc ranging from 100–120 ppm to their feed to obtain rapid development in chicks’ growth [16]. This increase in zinc leads to a rise in feed production costs and an increase in the Zn excretion in feces, which leads to environmental pollution. It affects the balance of other microelements and reduces vitamins. However, increasing the bioavailability of zinc may fix these problems, and zinc bioavailability increases when it is present in nano form. Recently, nano-zinc has been used as a feed additive because of the beneficial effects on the metabolism and health of birds. It improves immunity through its antibacterial activity [17,18,19]. Various studies stated that utilizing ZnNPs as a feed supplement enhances the following properties: body weight gain, feed conversion ratio, meat quality, and egg quantity. Additionally, it was also observed that it affected cecal microbiota and enhanced the immune system [20,21].

Curcumin has many pharmacological activities in treating various diseases [22]. It also increases the activity of digestive enzymes [22] and inhibits lipid oxidation [23]. Curcumin has antioxidant activity and controls the cecal microbiota. Curcumin alleviates oxidative liver injury by modulating the disruption of the cecum microbiota and lipid metabolism induced by ochratoxin A. Curcumin is recommended as a prophylactic measure to prevent ochratoxin A (OTA)-induced hepatic oxidative injury [24]. The nano form of curcumin increases its beneficial influences. It is easy for the nanoparticles to pass into the cell membranes and interact with the cell contents [25]. Hence, curcumin nanoparticles (CurNPs) increase the availability and the intake of curcumin [26]. A significant positive effect on chick’s performance was observed in the diet supplemented with CurNPs [27]. Sayrafi, et al. [28] stated that reducing liver enzyme activity after CurNPs addition might be due to its antioxidant properties. Moreover, Partovi, et al. [29] reported that supplementing 300 mg of CurNPs/kg diet was a useful nutritional source, which can improve carcass parameters, protein content, redness and oxidative stability of broiler chicken breast meat infected with *Eimeria* species and decrease drip loss and cooking loss while did not have negative effect on texture profile of the chicken broiler meat.

The use of probiotics in poultry diets has steadily increased across the years because of the highest demand for antibiotic-free poultry [30]. The probiotic market in 2018 had a profit of 80 million United States Dollar (USD) and the addition of probiotics in the poultry diet maintains the global probiotic market, as its profits are expected to reach 125 million USD by 2025, with an annual increase of 7.7% [30]. Probiotics in broiler diets increase the growth and laying outcomes, reduce pathogenic bacteria in the gut, and raise beneficial microbiota and immunity [31]. *Bacillus* sp. microbes are among the most extensively used, direct-fed growth promoters. These bacteria serve as an alternative to antibiotics. A broiler diet supplemented with *B. licheniformis* can significantly improve BWG and FCR despite *C. perfringens* infection [32,33]. These effects are mainly attributed to *B. licheniformis* can enhance nutrient digestion and utilization in broilers by producing several enzymes, such as lipase, protease, and amylase. Recently, Abou-Kassem, et al. [34] found that all of the growth and carcass aspects were significantly influenced by dietary probiotic addition compared to the control group in quail. Some previous studies have used single chemical and physical synthesized nanoparticle additives in broiler feed to assess their effects on broiler performance; other studies that included more than one feed additive reported synergistic effects due to their active components on different parameters of broiler performance [3,7]. Here, we hypothesized that the novel combination of biological synthesized nanoparticles and probiotics and their combinations might lead to promising and synergetic effects on most broiler performance traits. Thus, this study aimed to evaluate the antimicrobial activity of ZnNPs, CurNPs, and probiotic (*B. licheniformis*) and their synergetic effects on growth performance, carcass properties, blood indices, meat quality, and cecal microbial load.

## 2. Materials and Methods

### 2.1. Nanoparticles Biosynthesis and Antimicrobial Properties

#### 2.1.1. Bacterial Isolates, Biosynthesis and Characterization of ZnNPs and CurNPs

**Figure 1:**
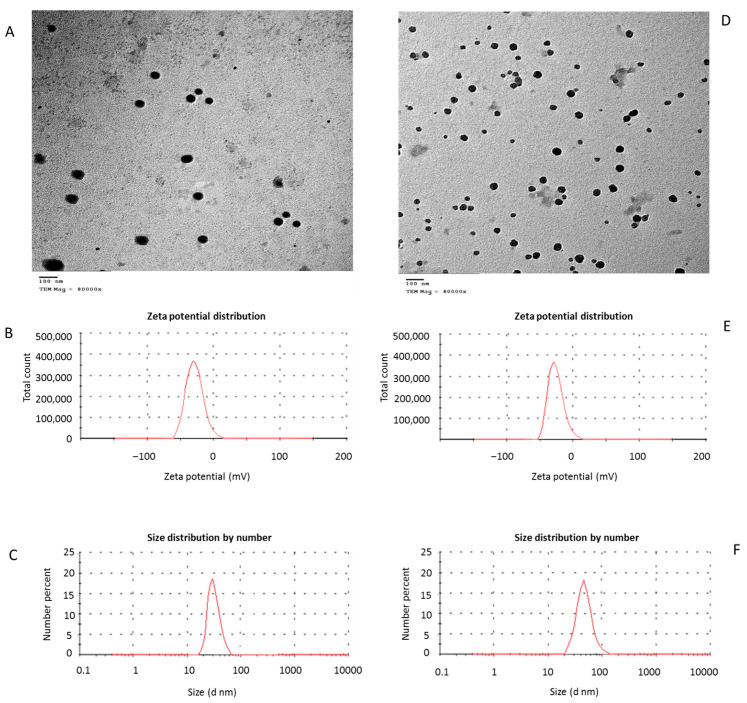
Characterization of zinc nanoparticles (ZnNPs) and curcumin nanoparticles (CurNPs); (**A**–**C**) shape, charge, and size of ZnNPs accessed by TEM, zeta potential, and zeta sizer, respectively. (**D**–**F**) shape, charge, and size of CurNPs accessed by TEM, zeta potential, and zeta sizer, respectively.

*Bacillus subtilis* LA4 and *Bacillus subtilis* AM12 were used in biosynthesize curcumin nanoparticles (CurNPs) and zinc nanoparticles (ZnNPs). These bacterial strains were isolated from soil samples collected from different regions in Zagazig city, Egypt [9,35,36,37]. The CurNPs were fabricated as follows, 40 mL of *Bacillus subtilis* LA4 supernatant was mixed with 60 mL of curcumin (0.27 mmol) in a 250 mL screw bottle then placed in a shaking incubator (160 rpm) at 30 °C for 72 h, pH 6, and nutrient broth media (NB) [25]. Other bottles containing 40 of NB and 60 mL of curcumin were incubated under the same conditions to prove the curcumin biotransformation occurred by *Bacillus subtilis* LA4 supernatant by the differences in color observation [25]. On the other hand, ZnNPs were produced by mixing 10 mL of *Bacillus subtilis* AM12 supernatant with 90 mL of zinc nitrate (1 mmol), pH 7, then was incubated in a shaking incubator (130 rpm) at 30 °C for 72 h. Zinc nitrate in NB was used as a control. Visible changes in the colorless mixture to white color indicate the biotransformation of zinc nitrate to ZnNPs by *Bacillus subtilis* AM12 supernatant [18]. Several methods were used to investigate the behavior of ZnNPs and CurNPs. The size, shape, and aggregation were assessed using transmission electron microscopy (TEM), the hydrodynamic size distribution was measured using dynamic light scattering (DLS), and the stability of the surface charge was estimated using zeta potential [38,39,40,41]. The obtained CurNPs were of spherical shape with a mean diameter of 65–80 nm measured by transmission electron microscopy (TEM) (JEOL 1010, JEOL Ltd., Tokyo, Japan), and had a negative charge of −25.3 mV by zeta potential analysis (Nano Z2 Malven, Malvern Hills, UK). The obtained ZnNPs were spherical with a mean diameter of 22–43 nm measured by TEM and a negative charge of −28.7 mV by zeta potential analysis (Figure 1).

#### 2.1.2. Antimicrobial Activity of CurNPs and ZnNPs

The bacterial isolates (Bacillus cereus, Listeria monocytogenes, and Staphylococcus pyogenes as gram-positive bacteria, Escherichia coli, Salmonella typhi, and Pseudomonas aeruginosa as gram-negative bacteria), and fungal isolates (Alternaria alternate, Aspergillus flavus, Fusarium oxysporum, Aspergillus niger, Penicillium solitum, and Penicillium crustosum) were used in this study to determine the antimicrobial activity of CurNPs and ZnNPs and obtained from Agricultural Microbiology department, Faculty of Agriculture, Zagazig University, Egypt. The antimicrobial activity of CurNPs and ZnNPs were estimated by the disc diffusion method [18,25,36]. Mueller Hinton agar (MHA) plates were inoculated with 0.1 mL of fresh bacterial inoculum and spread on the plate surface. Sabouraud dextrose agar (SDA) plates were inoculated with fungal mycelium disc in the plate center. The inoculated MHA and SDA plates were loaded with paper discs previously saturated with different concentrations of CurNPs and ZnNPs (100, 150, 200, 250, and 300 µg/mL). The paper discs were put on the sides of the plates. The MHA plates were incubated at 37 °C for a day and SDA plates at 28 °C for five days. The obtained inhibition zones diameters (mm) were measured [42,43].

The minimum inhibitory concentration (MIC) was estimated by the micro-dilution broth assay described in European Committee on Antimicrobial Susceptibility Testing [44]. In brief, tubes containing 9 mL of Muller Hinton broth (MHB) for bacteria or Sabourad dextrose broth (SDB) for fungi were inoculated with 0.1 mL of bacterial inoculum or standard fungal spore suspension (3 × 10^3^ CFU/mL), then 0.05 mL of CurNPs and ZnNPs at different concentrations (100, 150, 200, 250, and 300 µg/mL) were added. Free CurNPs and ZnNPs tubes were used as controls. The MHB and SDB tubes were incubated at 37 °C for a day and 28 °C for five days, respectively. The MIC was the lowest concentration of CurNPs and ZnNPs that inhibited bacterial and fungal growth. On the other hand, minimum bactericidal concentration (MBC) and minimum fungicidal concentration (MFC) were estimated according to CLSI [45]. A loop of MIC tubes were spread over new MHA or SDA plates and incubated at previous conditions, then observed the bacterial or fungal growth. The lowest concentration that kills the bacterial or fungal growth was considered (MBC) or (MFC) [46,47].

### 2.2. Birds, Experimental Design and Diets

This study was performed in Poultry Research Farm, Department of Poultry, Faculty of Agriculture, Zagazig University, Zagazig, Egypt. All procedures were carried out following the guidelines of the local committee for experimental animal care and confirmed by the ethics of the Institutional Council of the Poultry Department, Faculty of Agriculture, Zagazig University, Zagazig, Egypt. A total of 420 unsexed—one week old Indian River (IR) broilers with an initial body weight of 104.40 ± 0.12 g were used in a completely randomized design experiment involving seven groups with 60 birds per group six replicates of 10 animals each. Chicks were purchased from a commercial hatchery. Chicks were housed in floor pens with clean pine shavings-based litter (50 cm rise × 100 cm wide × 100 cm height; 10 chicks each) and exposed to near-continuous photoperiod length a 23 L:1D with rotation [48]. The ZnNPs, CurNPs (500 mg/L), and BL (1.5 × 10^8^ CFU/mL) were obtained from the Department of Agricultural Microbiology, Faculty of Agriculture, Zagazig University, Zagazig, Egypt and *B. licheniformis* was tested as probiotic bacteria [49]. The experimental groups were: the control group which was fed the basal diet. The first, second and third groups were given diets supplemented with ZnNPs, CurNPs and probiotics at 3.0, 5.0 and 2.0 mL/kg feed. The fourth, fifth and sixth groups were fed a diet supplemented with a combination of ZnNPs (3.0) + *B. licheniformis* (Bl) (2.0) (ZP); ZnNPs (3.0) + CurNPs (5.0) (ZC) and ZnNPs (3.0) + CurNPs (5.0) + *B. licheniformis* (BL) (2.0) (ZCP) mL/kg feed, respectively. Firstly, the nanoparticle of zinc and curcumin was added to the diet, then we did the pelletization process, after that, we top-dressed the probiotics on the pelletized feed to avoid the inactivation process of bacteria. The basal diets were formulated to meet the nutritional specifications of Indian River^®^ Broiler Management guide [50]. All chicks were given the diets in the pellets form for 1–5 weeks of age, as shown in Table 1. The diets were given in two stages: starter (1–3 weeks) and finisher (3–5 weeks). All chicks were managed in the same ecological, managerial and hygienic conditions.

### 2.3. Traits Measured

#### 2.3.1. Performance, Carcass, and Blood Biochemical Parameters

The parameters were measured once a week. Average day feed intake (FI), body weight gain (BWG), and feed conversion ratio (FCR) were calculated. Forty-two chicks were randomly selected from different pens within each treatment (one bird from each replicate within the group) and slaughtered at 35 days. The carcasses were weighed, and the edible parts (liver, gizzards, and hearts) and spleen, bursa, and abdominal fat were weighted as g/kg of the slaughter weight (SW). Carcass and dressed weights were expressed as (dressed weight = carcass weight + edible weight)/live body weight. The mortality rate was recorded weekly for each group and cumulatively calculated for the entire period of the experiment (1–5 weeks).

Blood sampling was performed during slaughtering from six birds randomly selected from different pens within each treatment (one bird from each replicate within the group). Samples were immediately centrifuged (Janetzki, T32c, 5000 rpm, Wall-hausen, Germany) at 2146.56× *g* for 15 min. The obtained serum was then frozen at −25 °C till the biochemical tests [51,52]. Hematological parameters (WBCs: white blood cells; LYM: lymphocytes; GRA: granulocytes; RBCs: red blood cells; HGB: hemoglobin; HCT: hematocrit; MCV: Mean corpuscular volume; MCH: Mean corpuscular hemoglobin; PLT: Platelet count) were measured according to Salvaggio et al. [53]. The levels of total protein, albumin, glucose, alkaline phosphatase (ALP), alanine amino-transferase (ALT), aspartate amino-transferase (AST), uric acid, creatinine, urea-N were measured as [53,54] triglycerides (TG), total cholesterol (TC), low-density lipoprotein (LDL), very-low-density lipoprotein (VLDL) and high-density lipoprotein (HDL) were measured using kits according to the protocol provided by the manufacturer (Spinreact, Ctra.Santa Coloma, Spain) [55]. Commercial kits from Biodiagnostic Company (Giza, Egypt) determined the immunity parameters IgG, A and M., antioxidant enzymes: glutathione (GSH), malondialdehyde (MDA). The activities of glutathione reductase (GSR), glutathione S-transferase (GST), superoxide dismutase (SOD), catalase (CAT) and glutathione peroxidase (GPx) were measured by a colorimetric method using kits according to the protocol provided by the manufacturer (Cell Biolabs Inc., San Diego, CA, USA).

#### 2.3.2. Breast Meat Quality and Sensory Evaluation

The color parameters [L* (lightness), a* (redness), and b* (yellowness)] of raw and cooked meat samples (cubes, 2 cm) (*n* = 6/treatment) were measured by Hunter Lab colorimeter (Color Flex EZ, Reston, VA, USA) following the procedure described in [56,57]. The shear force value of cooked meat cubes of 2 cm was measured by a texture analyzer (Compac-100 model, Sun Scientific Co., Tokyo, Japan) equipped with a cross head and a load cell. The speed of the cross head was set at 240 mm/min and a load cell of 10 kg was used. The cutting force was vertically applied to the meat fibers. The peak value profile of shear force was reported as the value of shear force. The lipid oxidation was measured by a 2-thiobarbituric acid test (TBA) [58]. Total volatile bases nitrogen (TVBN) was estimated according to Botta et al. [59]. The pH value of minced meat samples was assessed using a pH meter (pH 211 HANNA instruments Inc., Woonsocket, RI, USA). The chemical composition of meat was also estimated [57]. Moisture content was determined by oven method [60]; protein was determined by Kjeldahl method [60]; fat was estimated by the Soxhlet apparatus method [60]; a muffin assessed ash at 600 °C [60].

*Sensory evaluation*: the cooked meat samples (*n* = 6/treatment) were cut into cubes (2 cm) [61]. Eight experienced panelists have received meat samples in foam plate coded with random 3-digits. The sensory panel followed the descriptive sensory assessment carried out using a variation of the Sow and Grongnet [62] and Zhuang and Savage [63] process. The panelists have evaluated the following attributes (color, flavor, appearance, and juiciness) using a 7-point hedonic scale, where 1 = strongly dislike and 7 = strongly like. Tap water was provided between sessions to alter the mouth feel.

#### 2.3.3. Microbial Count in Diet and Cecal Samples

The dietary samples (*n* = 6/treatment) were microbiologically examined at intervals of 0, 7, 14, and 21 days. Dietary samples were mixed with sterile saline peptone water (1 g/L peptone and 8.5 g/L NaCl) at a screw bottle and homogenized for ten minutes. Different media were used to enumerate the microbial count. Total bacterial count (TBC) was counted at plate count agar after incubation at 30 °C for two days. The total yeasts and molds count (TYMC) were estimated on Rose Bengal Chloramphenicol agar after incubation for five days at 25 °C. Total coliforms were counted on Violet Red Bile Agar (Biolife, Italy) after incubation at 37 °C for 24 h [64]. *Escherichia coli* were counted at Tryptone Bile Glucuronide Agar after incubation at 37 °C for 24 h. Additionally, the microbial count in broiler cecum was estimated as in diet. Five cecal samples were obtained from each group randomly and then were homogenized in a sterilized screw bottle with sterile saline peptone water (1 g/L peptone and 8.5 g/L NaCl). Decimal serial dilutions up to 10^7^ were prepared. The different microorganisms in this study were counted on specific media [51,65,66,67]. The total bacterial count was enumerated as per Sheiha et al. [68] and Reda et al. [25] on Plate Count Agar (PCA) after incubation at 30 °C for two days. Violet Red Bile Agar (Biolife, Italy) was used for counting coliform after incubation at 37 °C for 24 h. *Escherichia coli* was counted at Tryptone Bile Glucuronide Agar after incubation at 37 °C for 24 h [69]. *Salmonella* spp. was counted on S.S. agar as per Edwards and Hilderbrand [70]. The yeasts and molds were enumerated as per Kurtzman et al. [71]. MRS-medium was used to count Lactic acid bacteria, according to Argyri et al. [72]. *Enterococcus* spp. was counted on Chromocult enterococci agar; red colonies indicated that it was found [73].

### 2.4. Statistical Analysis

The SPSS v 20 (IBM Corp., Armonk, NY, USA) and one-way ANOVA test were used to analyze the replicated data using GLM procedures. The statistical model used was:

Y_ij_ = µ + T_i_ + e_ij_ (1)

where Y_ij_ = observed value; µ = overall mean; T_i_ = treatment effect (control, and 1–6); and e_ij_ = random error. Differences among recorded means were estimated by the test of Student–Newman–Keuls. The SEM and mean values were reported. The differences between groups are considered significant at *p* < 0.05.

GLM model analyzed the data of the antimicrobial activity according to the following model:

Y_aijk_ = µ + M_a_ + N_i_ + C_j_ + N_i_*C_j_ + e_aij__k_ (2)

where Y_ijk_ = observed value; µ = overall mean; M_a_ = fixed effect of microorganism (bacteria or fungal), N_i_ = nanoparticle effect (zinc and curcumin), C_j_ = concentration of nanoparticles (100, 150, 200, 250 and 300 µg/mL for each nanoparticle type); N_i_*C_j_ = the interaction between nanoparticles and the dose of the nanoparticles; e_aijk_ = random error. The differences between means were compared by LSD at 5% level of probability.

GLM model analyzed the data of the microbiological activity of dietary samples at intervals according to the following model:

Y_ijk_ = µ + T_i_ + S_j_ + N_i_*S^j^ + e_ij__k_ (3)

where Y_ijk_ = observed value; µ = overall mean; N_i_ = treatment effect (control, and 1–6), S_j_ = day of sampling (0, 7, 14, and 21 days); T_i_*S_j_ = the interaction between treatment and the days of sampling; e_ijk_ = random error. The differences between means were compared by LSD at 5% level of probability.

## 3. Results

### 3.1. Antimicrobial Activity of CurNPs and ZnNPs

Table 2 showed the antibacterial effect of CurNPs and ZnNPs against six bacterial isolates. The inhibition zones diameters (IZDs) of CurNPs and ZnNPs were increasing in concentration-dependent manner. The results showed that CurNPs exhibited more antibacterial activity than ZnNPs; the CurNPs IZDs were in the range of 13.7–25.6 mm, the ZnNPs IZDs were in the range of 12.7–24.5. The largest IZD was observed against *S. pyogenes* with 25.6 and 24.5 for CurNPs and ZnNPs. Therefore, *S. pyogenes* was the most sensitive Gram-positive bacteria to the tested nanoparticles. On the other hand, *P. aeruginosa* was the most resistant Gram-negative bacteria to CurNPs and ZnNPs concentrations. Likely; the CurNPs had higher IZDs against tested fungi in a range of 17.9–30.2 mm compared to ZnNPs. *A. niger* was the most sensitive fungi to nanoparticles and *P. crustosum* was the most resistant. The CurNPs and ZnNPs inhibited the tested microbial growth with MIC values of 45–90 and 50–95 µg/mL, respectively. No microbial growth was detected at concentrations between 85–155, and 90–170 µg/mL for CurNPs, and ZnNPs. on the other hands, the combination between CurNPs and ZnNPs increased the antimicrobial activity achieving no bacterial or fungal growth in the range of 80–140 µg/mL (data not shown).

### 3.2. Growth Performance

The effects of CurNPs, ZnNPs and Bl on live body weight (LBW) and BWG of chicks are shown in Table 3. Tests of variance offered a remarkable (*p* < 0.001) impact of treatments on LBW, BWG and FI over the various experimental groups at the starter (1–3 weeks), finisher (3–5 weeks) and only effects on LBW and BWG at the whole cycle (1–5 weeks) compared to the control, respectively. For the whole cycle, the best LBW was for T5 and T6; and the best BWG was for all the groups with ZnNPs (T1, T4, T5 and T6).

The FI decreased significantly in all treated groups compared to control broilers at the starter period (1–3 weeks), while FI increased significantly in T1, T2, and T5 compared to other treated and control birds at the finisher period (3–5 weeks). On the other hand, FI did not exhibit any significant differences between all treated and control groups throughout the cycle (1–5 weeks) (Table 3).

The influences of biological ZnNPs, CurNPs and BL on FCR are illustrated in Table 3. Generally, diets supplemented with ZnNPs, CurNPs and *B. licheniformis* (Bl) gave a better FCR than the control. The T6 group showed the best FCR at the started period, whereas T3 and T4 presented the best FCR in the finisher period. Regarding the whole cycle T3, T4 and T6 presented the best FCR values.

### 3.3. Carcass Traits

As shown in Table 4, all carcass traits were significantly (*p* < 0.05) impacted by the dietary treatment except for % of the bursa. The best carcass and dressing values are for T6 and T4. However, the lowest value for carcass was for control, and the lowest for dressing was control and T2. All dietary treatments, except for T2 (increased), reduced abdominal fat percentage compared to control.

### 3.4. Blood Biochemical Indices

Compared to the control group; the dietary supplementation of CurNPs, ZnNPs, and *B. licheniformis* (Bl) combination in T5 and T6 significantly increased hemoglobin content, MCH, MCV, MCHC, RBCs count, monocytes %, albumen content and IgA level as well as GSH, GSR and SOD enzymes. T6 resulted in significant increase in PCV, blood platelets count, and percentages of lymphocytes, neutrophils, basophils, and eosinophils. Moreover, a significant increase in calcium level and GST, duodenal amylase, protease and lipase enzymatic activity was reported compared to the control group. Additionally, lipid indicators and parameters were significantly lower in the T6 group than in the control and other groups. A significant increase in SOD and GSH, immunoglobulins, and hydrolysis enzymes were observed in the T6 group compared with the control group and other groups (Table 5).

### 3.5. Meat Quality

Data in Table 6 reported that the addition of CurNPs, ZnNPs, and Bl (5:3:2 cm^3^/kg) to the broiler diet significantly increased meat moisture (T5 and T6), protein content (T6) and pH (T6) compared to control group. Moreover, the lipid content of meat was significantly lower in T6 compared to other treated and control groups. Furthermore, the T6 significantly enhanced the yellowness (b*), juiciness, tenderness and taste of the meat compared to the control group.

### 3.6. Microbial Count in Diet and Cecal Samples

Generally, the microbial count was significantly (*p* ≤ 0.05) lower in the treated groups than that of the control. However, the interaction effect showed that T6 excelled the other treatment groups in reducing the microbial count in diet samples with a relative decrease of 35% in total bacterial count (TBC), 45% in total yeasts and molds count (TYMC), 63% in *E. coli* count and 50% of coliform count compared to control. The microbial count increased with feeding period. The T6 followed T5 in the microbial count reduction (Table 7). Additionally, total bacterial count, total yeasts and molds, coliform, *E. coli*, *Salmonella* spp., *Enterococcus* spp., and lactic acid bacteria were counted in the cecum. Data in Table 8 showed that the T6 group followed by T5 caused a significant reduction in all microbial counts in the broiler’s cecum with a relative decrease of 18–30%. Conversely, the count of lactic acid bacteria was higher than the control by 25%. *Salmonella* did not exist in the cecum of the treated groups. The different treatments significantly reduced the count of *Enterococcus* spp.

## 4. Discussion

The antimicrobial and antioxidant activities of CurNPs and ZnNPs are attributable to their small size and their phenolic compounds content on the surface of nanoparticles (NPs) [74]. These active compounds may be the reason for the beneficial effects of NPs additive on the growth, carcass properties, biochemical blood indicators, meat quality and microbial status of birds and the combination between CurNPs and ZnNPs increased the antimicrobial and antioxidant activities. For the antimicrobial activity of curcumin nanoparticles against tested pathogenic bacteria, Bhawana, et al. [75] confirmed the antibacterial activity of CurNPs on *S. aureus, B. subtilis, E. coli,* and *p. aeruginosa*. The authors added that the effect of CurNPs was more effective on Gram-positive than Gram-negative bacteria. Additionally, Narayanan, et al. [76] reported that the IDZs of ZnONPs (40 μg/mL) against *S. aureus* and *E. coli* were 19 and 14 mm, respectively. The IDZ of ZnONPs (50 μg/mL) on these bacteria were 18 and 16 mm [77]. These results are in agreement with the obtained results. Sirelkhatim, et al. [78] and Hassani Sangani, et al. [79] showed that the MIC and MBC values of ZnONPs were in the range of 158–325 μg/mL against 15 isolates of *p. aeruginosa* as well. The MIC was 50 μg/mL against *E. coli*, *Bacillus subtilis*, and *Staphylococcus aureus* and 25 μg/mL against *Vibrio cholera* and *Clostridium botulinum*.

The CurNPs and ZnNPs exhibited broad-spectrum antifungal activity in the current study. Arciniegas-Grijalba, et al. [80] found that zinc oxide nanoparticles in vitro antifungal activity against *Erythricium salmonicolor* causal of pink disease was through inhibiting the fungal mycelia. Additionally, Alnashi and Fattah [81] revealed that curcumin nanoparticles produced by turmeric methanolic extract have antifungal activity against *Candida albicans* ATCC 10,231 and *Geotricum candidum* NRRL Y-552 and mold strains, including: *Aspergillus niger* ATCC 102, *Aspergillus flavus* ATCC 247 and *Fusarium moniliform* ATCC 206 with MIC range of 15–25µg/mL. No available studies discussed the combination between CurNPs and ZnNPs. The beneficial impacts of probiotics on bird’s performance may occur via changing the gut medium and raising the immunization of helpful gastric microorganisms. The competitive action reduces the harmful bacteria and the excitation of the immune system [31,82]. Probiotics settle the beneficial bacteria in the intestine and the competition with pathogenic bacteria leaves no area for hurtful bacteria to live in or set up. Additionally, probiotics stimulate the digestive enzyme secretion such as β galactosidase, α amylase, etc., which helps enhance the performance of animals [83].

The combinations of both NPs (T5) and the triple combination in the study (T6) presented the best LWG among groups, whereas all treatments supplemented with ZnNPs showed the best BWG values (T1, T4, T5 and T6) throughout the whole cycle. This positive effect may be due to the role of ZnNPs in raising the intestinal absorption ability by increasing the mucosal efficiency [84]. The higher adsorption capacity of Zn nanoparticles improves Zn bioavailability [21,85]. Previous studies indicated that utilizing ZnNPs as feed additives could improve LBW and FCR, reduce gut microbial populations, and boost the immunity system [20,21]. Our results agree with Mahmoud, et al. [86], who reported that ZnNPs (10 ppm) safely improved the body weight gain and FCR in broilers. As well, Fathi, et al. [87] observed that birds fed diets supplemented with nano-ZnO had higher (*p*< 0.05) body weight gain and lower FCR than the control. Several studies assured that diets supplemented with zinc increased growth rate and improved feed efficiency in broilers [21,88,89,90]. The different combinations between ZnNPs and Bl (T4), ZnNPs and CurNPs (T5), or ZnNPs, CurNPs, and Bl (T6) gave a reasonable growth rate compared to the control. This finding may depend on the role of each additive and their synergistic effect after combination. As reported in Table 3, the best growth performance (LBW, BWG, and FCR) was given by the T6 group, which had the triple combinations (ZnNPs, CurNPs, and Bl).

The nano form of curcumin increases its bioavailability and can raise its absorption [91], therefore, CurNPs can be applied as a safe and natural feed supplement [29]. The administration of CurNPs in chicks drinking water has also been reported to develop body weight and FCR. It has been reported that curcumin enhances the excretion of bile acids and stimulates protease, lipase, amylase, trypsin and chymotrypsin enzymes [92]. Therefore, the beneficial impact of curcumin on broiler growth may be due to the raised secretion of these enzymes. Recently, Reda et al. [25] showed that nano curcumin improved (*p* < 0.0001) LBW, BWG and FCR of growing quails at 5 and 1–5 weeks of age. The authors added that the FI (*p* < 0.0001) was reduced in birds fed CurNPs rations (0.1, 0.3, and 0.4 g/kg) compared to the control from 1 to 5 wks. The enhancements in the broiler performance given a diet supplemented with curcumin was probably due to improvements in the intestinal morphology of the broiler [27]. The considerable impact of curcumin might belong to its antibacterial, antioxidant and anti-inflammatory activities [93]. The method of using CurNPs in broilers’ diets has been recently examined. It may have a potential pathway to activate the physiological and health status of broilers.

For carcass traits, the enhancement in some carcass traits in birds received T6 diet may be attributed to the antimicrobial activity of ZnNPs that reduces the pathogenic microbes’ load and improves gut health [94]. Previous investigations showed that dietary ZnNPs (40 to 90 ppm) supplementation increased dressing percentage and carcass yield [5,93,95]. Moreover, Mahmoud et al. [86] confirmed that relative weights of spleen and bursa in birds fed diets supplemented with 10, 20, or 40 ppm of ZnNP’s were higher (*p* < 0.05) than the untreated groups. However, Abdel-Moneim et al. [96] found no statistical differences in carcass traits of broilers fed *bifidobacteria* administrated groups.

The results obtained from Table 5, Table 6, Table 7 and Table 8 showed an essential impact for feed supplementation concerning the biochemical indices, enzyme secretion, immunity status, increasing beneficial microorganisms, and reducing pathogenic bacteria, that achieving the best health and performance. No available studies investigate the beneficial impact of a similar combination; however, the findings of the current study were similar to the obtained results in a single addition Premavalli, et al. [97] and Abdel-Moneim, et al. [98] on growing Japanese quail and Jin, et al. [99] and Abd El-Moneim et al. [96] on broilers. The latter authors showed the valuable action of probiotics on bird’s viability and healthy digestive tract. Zinc oxide nanoparticles can impact the birds’ metabolic potency and health status due to their anti-bacterial and immune-modulation properties [18,19]. In addition, various studies revealed that higher dosages of ZnNPs such as 30–80 ppm [20,90] could enhance the broiler performance [85]. This is because zinc is an important microelement and a portion of more than 300 enzymes participating in controlling nucleic acid and protein metabolism [100,101]. Furthermore, ZnNPs can adjust the broiler’s metabolism by raising the actions of insulin and growth hormone genes [102]. El-Katcha, et al. [103] mentioned that dietary addition of ZnNPs at 15 ppm enhanced the weight gain of broilers. In line, Zhao et al. [21] found that ZnNPs have beneficial effects on enhancing broiler performance.

The increasing in immunoglobulin levels in blood and cells in T6 may be attributed to synergistic effects of the triple combinations of ZnNPs, CurNPs and *Bacillus licheniformis* through the additivity of their antioxidant properties that resulted in the enhancement of the immune systems of birds. The antioxidant activity of CurNPs has a modulating effect on blood indices. The addition of CurNPs to the broiler’s diet lowered AST levels in the blood [28]. Conversely, increased LDH levels and improved liver functions were reported in broilers fed a diet supplemented with turmeric (5 g/kg) [104]. Previous reports confirmed that feed supplemented with CurNPs (400 mg/kg diet) significantly decreased the lipid profile and reduced blood cholesterol as a diagnostic marker of lipid metabolism [105,106,107]. Emadi and Kermanshahi [104] observed a decrease in HDL levels and increased LDL levels in chickens that consumed a diet supplemented with turmeric. This led to improved liver function because of the inhibition of the HMGCR enzyme responsible for TC production in hepatic tissues [108]. Curcumin is a powerful antioxidant [109]. It reduces oxidative stress by modifying hepatic nuclear transcription factors and reducing lipid peroxidation in serum and muscles [110].

Diet supplemented with turmeric roots increased the levels of SOD, GSH and decreased levels of MDA [111]. Dietary curcumin supplementation reduced blood MDA and increased CAT, SOD, and GSH levels compared to controls [110,112]. It has been proven that the turmeric plant activates the immune cells (B and T) [113]. On the other hand, the addition of ZnNPs to broiler’s diet led to an increase in the levels of blood lipids according to Fathi et al. [87]; Al-Daraji and Amen [114]. The latter authors also stated that a diet supplemented with ZnNPs (20 mg/kg) led to increased blood cholesterol. This increase is due to the role of zinc as the main part of many lipid enzymes. However, it stimulates SOD activity to scavenge free radicals [21]. Probiotics did not affect serum parameters except serum calcium and glucose Alkhalf, et al. [115].

The obtained results showed that ZnNPs, CurNPs, and Bl combinations gave the best improvements in meat quality. It increased the moisture and protein content and increased the tenderness and juiciness of the meat. Likewise, adding some natural extracts such as green tea extract and grape seeds to cooked beef patties did not affect the meat’s sensory properties [116,117]. Additionally, the triple combination had an increased pH value that may differ due to the basic nature of the combination among ZnNPs, CuNPs, and Bl. However, TBVN and TBA values were significantly decreased, indicating the inhibitory effect of this combination against lipid oxidation and protein deterioration of meat by microbial enzymes [21].

The intestinal microbial load plays an important role in poultry health. The addition of CurNPs to the broiler diet modified the microbial balance in the intestine by increasing lactic acid bacteria count and reducing the pathogenic bacteria count, i.e., *S. aureus* and *E. coli* compared to the control [118,119]. Gupta, et al. [120] found that turmeric extracts inhibit pathogenic bacteria. Moreover, they reduce the intestinal bacterial count [121]. On the other hand, the diet supplemented with biological ZnNPs (100 mg/kg) caused increased bacterial count except for *E. coli* and *Enterococcus* spp. While the dietary addition of 200 and 400 mg ZnNPs/kg diet significantly decreased the bacterial count except for the intestinal bacteria. The concentration of 300 mg ZnNPs/kg diet significantly increased the bacterial load except for *Salmonella* spp.

The combination of (ZnNPs^+^ CurNPs^+^ Bl) achieved synergism, and their production was economic-effective. The synergism resulted in improving growth performance, blood indices, meat quality, and antioxidants parameters. The combination between CurNPs, ZnNPs, and BL exhibited more antimicrobial activity than the individual additions. Sequentially, the pathogenic microorganism reduced and increased lactic acid bacteria compared to single additions. Conclusively, the cost of production of biologically-synthesized CurNPs and ZnNPs is very low compared to the other methods (chemical or physical) with higher safety levels. In our study, the nanoparticles were used in very small doses compared to the original additives of the curcumin and zinc in broiler feed used in higher doses, which is very cost-effective. Additionally, BL is considered a very safe and cheap probiotic compared to other feed additives included in broiler feed, such as antibiotics.

## 5. Conclusions

The results of the current study assured a considerable antimicrobial activity against pathogenic bacteria and fungi with ZnNPs and CurNPs supplementation. The combination of (ZnNPs+ CurNPs+ Bl) achieves synergistic effects on enhancing the broiler’s weights, performance, carcass traits, digestive enzymes, meat quality traits, blood indices, and cecal microbial load and the antioxidants capacity. Therefore, the inclusion of ZnNPs, CurNPs, Bl, and their combinations is recommended for broiler feeding regimens to improve the performance and health status with economic benefits.

## Figures and Tables

**Table 1:** Composition and chemical analysis of the starter and finisher basal diets (as fed).

Items	Starter (1–3 Weeks)	Finisher (3–5 Weeks)
Ingredients %		
Yellow corn	55.89	57
Soybean meal 44%	31.5	29.5
Gluten meal 60%	6.5	4.83
Dicalcium phosphate	1.7	1.7
Limestone	1.24	1.15
Vit-min Premix *	0.3	0.3
NaCl	0.3	0.3
DL-Methionine	0.13	0.0
L-Lysine HCl	0.24	0.18
Choline 50%	0.2	0.2
Soybean oil	2.0	4.84
Total	100	100
Calculated analysis:		
Dry matter %	91.72	90.43
Crude protein %	23.00	20.94
Metabolizable energy (kcal/kg diet)	2996.30	3150.70
Calcium %	1.00	0.96
Phosphorous (Available) %	0.44	0.44
Lysine %	1.3	1.17
Methionine + Cysteine %	0.90	0.70
Crude fiber %	3.52	3.38

* Vitamin-mineral premix provided per kg diet: vitamin A, 12,000 IU; vitamin D_3_, 5000 IU; vitamin. E, 16.7 g; vitamin K, 0.67 g; vitamin B_1_, 0.67 g; vitamin B_2_, 2 g; vitamin B_6_, 0.67 g; vitamin B_12_, 0.004 g; nicotinic acid, 16.7 g; pantothenic acid, 6.67 g; biotin, 0.07 g; folic acid, 1.67 g; choline chloride, 400 g; Zn, 23.3 g; Mn, 10 g; Fe, 25 g; Cu, 1.67 g; I, 0.25 g; Se, 0.033 g and Mg, 133.4 g.

**Table 2:** Antimicrobial activity of ZnNPs and CurNPs against the tested bacteria and fungi represented by the inhibition zones diameters (mm), MIC, MFC and MBC (µg/mL). (*n* = 6).

Microorganisms	ZnNPs (µg/mL)					Means	CurNPs (µg/mL)					Means	MIC		MBC	
Bacteria	100	150	200	250	300		100	150	200	250	300		ZnNPs	CurNPs	ZnNPs	CurNPs
*B. cereus*	17.26 b	18.22 b	20.91 b	21.65 b	22.84 b	20.18 ^AB^	18.32 b	19.62 b	21.41 b	22.22 b	23.43 b	21.00 ^AB^	60.12 d	55.23 d	110.00 e	100.31 e
*L. monocytogenes*	16.55 c	17.53 c	19.52 c	20.54 c	23.42 c	19.5 ^B^	17.43 c	18.93 c	20.95 c	23.43 c	24.22 c	20.99 ^B^	75.21 cd	69.24 cd	130.01 d	120.21 d
*S. pyogenes*	18.36 a	19.45 a	21.83 a	22.92 a	24.53 a	21.42 ^A^	19.55 a	20.34 a	22.53 a	23.65 a	25.64 a	22.34 ^A^	50.00 e	45.33 e	90.23 f	85.43 f
*E. coli*	15.28 d	16.96 d	18.81 d	19.57 d	20.67 d	18.26 ^BC^	16.28 d	18.38 d	19.24 d	20.56 d	21.48 d	19.19 ^C^	80.13 c	71.11 c	140.14 c	130.24 c
*S. typhi*	14.38 e	15.28 e	17.24 e	18.48 e	19.88 e	17.05 ^C^	15.29 e	16.97 e	18.56 e	19.41 e	20.69 e	18.18 ^CD^	85.36 b	80.10 b	150.62 b	140.78 b
*p. aeruginosa*	12.79 f	14.82 f	16.46 f	17.55 f	18.52 f	16.03 ^D^	13.41 f	15.59 f	17.44 f	18.22 f	19.52 f	16.84 ^D^	95.61 a	90.34 a	170.14 a	160.14 a
*Means*	15.77 ^D^	17.04 ^C^	19.13 ^B^	20.12 ^AB^	21.64 ^A^		16.71 ^D^	18.31 ^C^	20.02 ^B^	21.25 ^AB^	22.50 ^A^		74.41 ^A^	68.56 ^B^	131.86 ^A^	122.85 ^B^
*SEM*	0.23	0.56	0.37	0.67	0.50		0.36	0.44	0.40	0.70	0.49		0.56	0.60	0.88	0.99
*p*-value																
Bacteria (B)			<0.001						<0.001				<0.001	<0.001	<0.001	<0.001
Concentration (C)			<0.001						<0.001							
B × C			<0.001						<0.001							
**Fungi**						Means						Means	MIC		MFC	
*A. alternate*	20.91 c	21.51 c	22.92 c	24.21 c	25.25 c	22.96 ^B^	21.42 c	22.24 c	23.54 c	25.02 c	26.42 c	23.73 ^B^	70.12 c	65.33 c	120.23 d	110.24 d
*A. flavus*	21.81 b	22.32 b	24.23 b	26.52 b	28.22 b	24.62 ^AB^	22.32 b	23.05 b	24.82 b	27.33 b	29.42 b	25.39 ^AB^	65.13 cd	60.27 cd	110.24 e	105.34 e
*F. oxysporum*	19.62 d	20.55 d	21.94 d	23.24 d	24.23 d	21.92 ^C^	20.13 d	21.22 d	22.55 d	24.05 d	25.43 d	22.68 ^BC^	75.52 bc	70.31 bc	140.29 c	130.24 c
*A. niger*	22.43 a	24.84 a	25.79 a	27.65 a	29.14 a	25.97 ^A^	22.95 a	25.52 a	26.38 a	28.46 a	30.24 a	26.71 ^A^	55.15 d	50.51 d	100.23 f	90.34 f
*p. solitum*	18.52 e	19.57 e	20.88 e	22.67 e	23.42 e	21.01 ^C^	19.06 e	20.21 e	21.42 e	23.47 e	24.68 e	21.77 ^C^	80.33 b	75.36 b	150.14 b	140.22 b
*p. crustosum*	16.43 f	18.22 f	19.54 f	21.48 f	22.61 f	19.66 ^D^	17.92 f	18.93 f	20.11 f	22.22 f	23.87 f	20.61 ^D^	95.56 a	90.25 a	170.34 a	155.31 a
**Means**	19.95 ^D^	21.17 ^C^	22.55 ^B^	24.30 ^AB^	25.48 ^A^		20.63 ^D^	21.86 ^C^	23.14 ^B^	25.09 ^AB^	26.68 ^A^		73.64 ^A^	68.67 ^B^	131.91 ^A^	121.95 ^B^
*SEM*	0.25	0.60	0.33	0.74	0.43		0.32	0.41	0.43	0.77	0.40		0.51	0.62	0.90	0.83
*p*-value																
Fungi (F)			<0.001						<0.001				<0.001	<0.001	<0.001	<0.001
Concentration (C)			<0.001						<0.001							
F × C			<0.001						<0.001							

a–f and ^A^^–^^D^ Mean in the same column within bacterial or fungal isolates within the same nanoparticle concentration with different lowercase letters are significantly different at *p* < 0.05; differences between means of the same bacterial or fungal isolates within the row and same concentration of different nanoparticles considered significant at *p* < 0.05; MIC: minimum inhibitory concentration; MBC: minimum bactericidal concentration; MFC: minimum fungicidal concentration. SEM: standard errors means.

**Table 3:** Live body weight (g), body weight gain (g), feed intake (g), feed conversion ratio and mortality rate (%) of broilers as affected by dietary supplementation of ZnNPs, CurNPs, and *Bl*. (*n* = 60/treatment).

Items	Treatments							SEM	*p*-Value
	Control	1	2	3	4	5	6		
Starter period (1–3 weeks)									
LBW (g)	667.41 b	642.36 d	645.36 d	652.45 c	668.18 b	665.95 b	683.73 a	4.03	0.038
BWG (g/day)	40.53 a	36.40 b	33.15 c	35.04 bc	38.02 b	37.25 b	40.12 a	0.59	<0.001
FI (g/day)	70.74 a	55.42 b	53.53 b	54.13 b	56.87 b	57.52 b	58.08 b	1.33	<0.001
FCR	1.74 a	1.52 c	1.61 b	1.55 c	1.50 c	1.54 c	1.45 d	0.02	0.004
Finisher period (3–5 weeks)									
LBW (g)	1843.30 e	2161.91 b	2062.36 de	2084.15 d	2143.43 c	2177.60 a	2190.54 a	26.26	<0.001
BWG (g)	81.05 c	117.61 a	112.38 ab	109.27 b	112.30 ab	118.72 a	114.54 ab	2.74	<0.001
FI (g)	116.48 d	148.56 a	150.57 a	131.66 c	136.11 c	150.47 a	141.66 b	3.13	0.007
FCR	1.44 a	1.26 c	1.34 b	1.20 d	1.21 d	1.27 c	1.24 c	0.02	0.034
Whole cycle (1–5 weeks)									
LBW (g)	1843.30 e	2161.91 b	2062.36 de	2084.15 d	2143.43 c	2177.60 a	2190.54 a	26.26	<0.001
BWG (g/day)	56.08 c	65.22 a	61.69 b	63.05 b	64.87 a	65.52 a	66.28 a	0.78	<0.001
FI (g/day)	87.69	89.84	84.65	84.02	86.88	90.63	88.31	0.75	0.133
FCR	1.56 a	1.39 b	1.40 b	1.35 c	1.35 c	1.39 b	1.33 c	0.02	0.001
Mortality rate (%)	2.30	2.72	2.72	0.00	1.82	2.72	0.00	0.82	0.542

Basal diet (Control)**;** T1: basal diet + 3.0 cm^3^ ZnNPs; T2: basal diet + 5.0 cm^3^ CurNPs; T3: basal diet + 2.0 cm^3^ BL; T4: basal diet + 3.0 cm^3^ ZnNPs + 2 cm^3^ BL; T5: basal diet +3.0 cm^3^ ZnNPs + 5.0 cm^3^ CurNPs and T6: basal diet +3.0 ZnNPs + 5.0 CurNPs + 2.0 cm^3^ BL. Live body weight (LBW); body weight gain (BWG); feed intake (FI); feed conversion ratio (FCR). SEM: standard error mean. a–e, Different letters within one row are significantly different (*p* < 0.05).

**Table 4:** Carcass traits (%) of broilers as affected by dietary supplementation of ZnNPs, CurNPs and *Bl*. (*n* = 6).

Carcass Traits (as a % of Pre-Slaughter Weight)							Items
Bursa	Spleen	Abdominal Fat	Dressing	Giblets	Carcass	Pre-Slaughter Weight	
0.10	0.12 b	1.11 b	77.29 d	3.28 e	74.00 d	2010.00 bc	Control
0.12	0.08 c	0.81 d	78.69 b	4.20 a	74.49 c	2047.50 b	T1
0.08	0.09 c	1.32 a	77.51 d	3.88 b	73.63 e	1960.00 c	T2
0.14	0.12 b	0.38 e	78.85 b	3.76 c	75.09 b	1995.00 bc	T3
0.13	0.16 a	0.94 c	79.19 ab	3.60 d	75.58 a	2240.00 a	T4
0.15	0.12 b	0.84 d	78.08 c	3.76 c	74.32 c	2020.00 bc	T5
0.17	0.12 b	0.79 d	79.55 a	3.71 c	75.85 a	2075.00 b	T6
0.01	0.01	0.07	0.23	0.11	0.71	21.45	SEM
0.135	0.017	0.002	0.006	<0.001	0.010	0.001	*p*-value

Basal diet (Control); T1: basal diet + 3.0 cm^3^ ZnNPs; T2: basal diet + 5.0 cm^3^ CurNPs; T3: basal diet + 2.0 cm^3^ BL; T4: basal diet + 3.0 cm^3^ ZnNPs + 2 cm^3^ BL; T5: basal diet + 3.0 cm^3^ ZnNPs + 5.0 cm^3^ CurNPs and T6: basal diet + 3.0 ZnNPs + 5.0 CurNPs + 2.0 cm^3^ BL. SEM: standard error mean. a–e Different letters within one column are significantly different (*p* < 0.05).

**Table 5:** Blood hematology, biochemical parameters, serum antioxidants, duodenal enzymes and immunity of broilers as affected by dietary supplementation of ZnNPs, CurNPs, and *Bacillus licheniformis*. (*n* = 6).

Serum Parameters	Control	T1	T2	T3	T4	T5	T6	SEM	*p* Value
Hematological									
Hemoglobin (g/dL)	6.90 bc	7.00 b	7.20 ab	7.00 b	7.00 b	7.20 ab	7.30 a	0.051	<0.0001
MCH (P/g)	22.90 c	23.00 bc	23.80 b	23.00 bc	22.90 c	24.00 ab	25.10 a	0.279	<0.0001
MCV (Fl)	63.00 d	63.50 c	64.00 bc	63.80 c	64.50 b	65.00 ab	66.50 a	0.405	<0.0001
MCHC (g/d)	33.22 cd	33.90 c	34.50 b	34.00 bc	33.50 c	35.10 ab	35.50 a	0.285	<0.0001
PCV (%)	22.25 c	22.50 bc	25.20 ab	21.50 cd	23.00 b	24.50 b	25.90 a	0.579	<0.0001
RBC (×10^6^/U L)	3.30 b	3.40 b	3.60 ab	2.50 c	3.40 b	3.50 ab	3.80 a	0.144	<0.0001
WBC (×10^3^ j/L)	4.00 a	4.00 a	3.25 c	2.10 d	3.50 b	3.12 bc	4.10 a	0.251	<0.0001
Platelets (10^3^ j/L)	210 cd	212 c	215 bc	199 e	219 b	205 d	225 a	3.027	<0.0001
Lymphocytes (%)	72.00 bc	67.10 d	73.10 b	72.10 bc	72.12 bc	70.31 c	75.12 a	0.877	<0.0001
Neutrophils (%)	21.00 d	21.10 d	27.12 b	25.60 c	29.12 ab	23.10 cd	30.12 a	1.286	<0.0001
Basophils (%)	0.00	0.00	0.00	1.10 b	1.20 b	0.90 bc	1.50 a	0.228	<0.0001
Monocytes (%)	0.30 d	0.40 d	1.90 bc	0.90 c	2.30 b	2.50 ab	2.80 a	0.363	<0.0001
Eosinophils (%)	4.30 b	4.50 ab	3.80 c	4.30 b	4.50 ab	4.10 c	5.13 a	0.135	<0.0001
Biochemical									
Glucose (mmol/L)	6.12 bc	6.11 bc	6.22 bc	6.30 ab	6.10 b	6.50 a	6.01 bc	0.069	<0.0001
Total protein (g/dL)	4.50 d	4.90 d	5.80 b	6.50 a	5.12 c	5.10 c	6.30 ab	0.266	<0.0001
Albumin (g/dL)	3.20 cd	3.50 c	4.10 b	4.10 b	4.00 b	4.20 ab	4.50 a	0.153	<0.0001
Globulin (g/dL)	2.10 d	2.50 c	2.60 c	2.40 cd	2.50 c	3.05 b	3.60 a	0.171	<0.0001
Chloride (mmol/L)	105 a	99 d	95 d	100 c	103 b	100 c	90.00 e	1.755	<0.0001
Calcium (mmol/L)	2.20 c	2.40 c	3.50 a	3.20 ab	3.05 b	3.05 b	3.50 a	0.177	<0.0001
AST (U/I)	23.12 c	25.42 bc	25.12 bc	35.12 a	27.05 b	34.22 ab	20.10 c	1.948	<0.0001
ALT (U/I)	20.14 c	16.10 d	19.01 c	25.22 ab	23.23 b	26.14 a	18.10 cd	1.309	<0.0001
Urea (mmol/L)	5.12 a	4.20 b	3.90 c	5.01 a	4.25 b	3.99 c	4.10 bc	0.161	<0.0001
Triglycerides (mmol/L)	1.20 a	1.10 ab	0.90 c	1.05 b	1.01 b	0.98 bc	0.70 c	0.056	<0.0001
Cholesterol (mmol/L)	4.10 ab	4.20 a	4.05 b	4.20 a	4.04 c	4.15 bc	4.12 c	0.030	<0.0001
LDL (mmol/L)	3.40 ab	3.50 a	1.70 c	3.40 ab	1.70 c	2.40 b	1.60 c	0.310	<0.0001
VLDL (mmol/L)	0.44 ab	0.45 a	0.30 b	0.28 c	0.17	0.32 ab	0.25 c	0.035	<0.0001
Oxidative enzymes									
GSH	1.10 d	1.30 c	1.30 c	1.20 cd	1.50 b	1.70 ab	1.80 a	0.091	<0.0001
GSR	1.30 d	1.50 cd	1.60 c	1.35 cd	1.80 b	1.90 ab	2.00 a	0.095	<0.0001
GST	1.40 cd	1.60 c	1.60 c	1.31 cd	1.80 b	2.00 b	2.30 a	0.121	<0.0001
SOD	1.50 e	1.70 d	1.80 cd	1.90 c	2.40 b	2.50 ab	2.70 a	0.160	<0.0001
MDA	17.90 a	17.10 ab	16.80 b	16.20 bc	16.20 bc	14.50 c	13.10 d	0.575	<0.0001
Duodenal Enzyme activity									
Amylase	2830 g	2935 f	3040 e	3145 d	3750 c	4022 b	4155 a	192.42	<0.0001
Protease	130 fg	140 f	170 e	180 d	190 c	225 b	234 a	13.72	<0.0001
Lipase	95.00f	100 e	111 de	120 d	130 c	140 b	145 a	6.75	<0.0001
Immunoglobulin level									
IgA	7.50 cd	7.80 c	7.90 bc	8.00 bc	8.50 b	9.70 ab	10.20 a	0.362	<0.0001
IgM	2.50 cd	2.90 c	3.00 cd	3.10 cd	3.50 c	3.90 b	4.50 a	0.237	<0.0001
IgG	13.00 cd	14.30 c	15.90 bc	16.00 bc	16.00 bc	17.00 b	18.90 a	0.658	<0.0001

Basal diet (Control); T1: basal diet + 3.0 cm^3^ ZnNPs; T2: basal diet + 5.0 cm^3^ CurNPs; T3: basal diet + 2.0 cm^3^ Bl; T4: basal diet + 3.0 cm^3^ ZnNPs + 2 cm^3^ BL; T5: basal diet + 3.0 cm^3^ ZnNPs + 5.0 cm^3^ CurNPs and T6: basal diet + 3.0 ZnNPs + 5.0 CurNPs + 2.0 cm^3^ Bl. MCH: mean corpuscular hemoglobin; MCV: mean corpuscular volume; MCHC: mean corpuscular hemoglobin concentration; PCV: packed cell volume; RBCs: red blood cells; WBCs: white blood cells; AST: aspartate aminotransferase; ALT: alanine aminotransferase; LDL: low-density lipoprotein; VLDL: very low-density lipoprotein; GSH: glutathione; GSR: glutathione reductase; GST: glutathione-S-transferase; SOD: superoxide dismutase; MDA: malondealdehyde. IgA: immunoglobulin A; IgM: immunoglobulin M; IgG; immunoglobulin G. SEM: standard error mean. a–g Different letters within one raw are significantly different (*p* < 0.05).

**Table 6:** Chemical, color parameters, and raw and cooked (sensorial) meat quality of broiler affected by dietary supplementation of ZnNPs, CurNPs, and *Bacillus licheniformis*. (*n* = 6).

Quality Parameters	Control	T1	T2	T3	T4	T5	T6	SEM	*p*-Value
Chemical									
Moisture	65.9 cd	64.30 d	68.00 b	68.20 b	67.10 c	70.00 ab	71.00 a	0.803	<0.0001
Protein	19.45 d	20.12 c	21.40 c	22.00 b	21.20 c	21.22 c	23.00 a	0.408	<0.0001
Lipid	14.1 ab	15.00 a	10.20 c	9.30 cd	11.50 b	9.00 d	6.00 e	1.087	<0.0001
Ash	0.89 b	1.10 a	0.90 b	1.00 ab	1.00 ab	0.80 c	0.30 d	0.092	<0.0001
pH	5.5 de	6.00 c	5.80 d	6.10 c	6.30 b	6.20 bc	6.80 a	0.143	<0.0001
TBVN	6.4 a	5.90 b	5.90 b	5.60 bc	5.50 bc	5.10 c	4.80 cd	0.187	<0.0001
TBA	0.60 a	0.60 a	0.5 ab	0.4 b	0.3 b	0.2 c	0.2 c	0.061	<0.0001
Color									
L*	60.10 ab	58.20 c	60.20 ab	60.00 ab	59.60 b	59.0 bc	61.20 a	0.334	<0.0001
a*	6.00 bc	6.50 ab	6.00 bc	6.00 bc	6.70 a	6.40 b	5.80 c	0.116	<0.0001
b*	15.00 bc	15.00 bc	15.90 b	14.10 cd	14.80 c	14.20 cd	16.10 a	0.268	<0.0001
Sensorial									
Juiciness	4.20 bc	4.05 c	4.35 b	4.30 b	4.4 b	4.56 ab	4.80 a	0.085	<0.0001
Tenderness	4.90 b	4.75 bc	4.70 c	4.95 ab	4.70 c	4.96 ab	5.20 a	0.063	<0.0001
Taste	4.35 c	4.20 cd	4.44 b	4.34 c	4.20 cd	4.56 ab	4.80 a	0.075	<0.0001
Aroma	4.50 ab	4.35 c	4.50 ab	4.55 ab	4.56 ab	4.46 b	4.70 a	0.037	<0.0001

Basal diet (Control)**;** T1: basal diet + 3.0 cm^3^ ZnNPs; T2: basal diet + 5.0 cm^3^ CurNPs; T3: basal diet + 2.0 cm^3^ Bl; T4: basal diet + 3.0 cm^3^ ZnNPs + 2 cm^3^ BL; T5: basal diet + 3.0 cm^3^ ZnNPs + 5.0 cm^3^ CurNPs and T6: basal diet + 3.0 ZnNPs + 5.0 CurNPs + 2.0 cm^3^ Bl; TVBN: total volatile basic nitrogen; TBA: thiobarbituric acid; L*: lightness; a*: redness; b*: yellowness. SEM: standard error mean. a–e Different letters within one raw are significantly different (*p* < 0.05).

**Table 7:** Dietary microbiota (total bacteria, yeast and molds, *E. coli*, and coliform) presented (Log CFU/mL) in broiler during feeding period of 0–21 days as affected by dietary supplementation of ZnNPs, CurNPs, and *Bacillus licheniformis*. (*n* = 6).

Samples/Microbial Count	TBC				*p* Value	TYMC				*p* Value
Feeding Period (day)	0	7	14	21		0	7	14	21	
Control	5.80 a,D	6.01 a,C	6.33 a,B	6.70 a,A	<0.001	3.81 a,D	4.00 a,C	4.30 a,B	4.82 a,A	<0.001
T1	5.54 ab,D	5.72 b,C	6.12 ab,B	6.51 ab,A	<0.001	3.52 ab,D	3.72 ab,C	4.03 ab,B	4.55 b,A	<0.001
T2	5.20 bD	5.41 bcC	5.93 bB	6.22 b,A	<0.001	3.14 b,D	3.51 b,C	3.82 b,B	4.12 bc,A	<0.001
T3	4.86 c,D	5.14 c,C	5.50 bc,B	5.90 bc,A	<0.001	2.82 bc,D	3.14 c,C	3.44 c,B	3.84 c,A	<0.001
T4	4.60 d,D	4.95 cd,C	5.32 c,B	5.74 c,A	<0.001	2.58 c,D	2.90 cd,C	3.15 cd,B	3.69 cd,A	<0.001
T5	4.22 d,D	4.72 d,C	5.11 cd,B	5.38 cd,A	<0.001	2.30 cd,D	2.71 d,C	2.82 d,B	3.33 d,A	<0.001
T6	3.93 e,A	4.11 e,C	4.85 d,B	5.09 d,A	<0.001	2.10 d,D	2.42 de,C	2.68 de,B	3.15 de,A	<0.001
*SEM*	0.25	0.27	0.61	0.44		0.46	0.64	0.09	0.14	
*p* value	<0.001	<0.001	<0.001	<0.001		<0.001	<0.001	<0.001	<0.001	
**Samples/Microbial Count**	***E. coli***				***p*** **Value**	**Coliform**				***p*** **Value**
**Feeding Period (day)**	**0**	**7**	**14**	**21**		**0**	**7**	**14**	**21**	
Control	2.22 a,C	2.56 a,B	2.92 a,AB	3.12 a,A	<0.001	2.91 a,D	3.33 a,C	3.62 a,B	4.02 a,A	<0.001
T1	2.01 ab,D	2.32 ab,C	2.62 ab,B	2.81 ab,A	<0.001	2.72 ab,D	3.05 ab,C	3.43 ab,B	3.81 ab,A	<0.001
T2	1.88 b,D	2.11 b,C	2.31 b,B	2.54 b,A	<0.001	2.44 b,D	2.84 b,C	3.11 b,B	3.51 b,A	<0.001
T3	1.56 bc,D	1.92 c,C	2.15 bc,B	2.37 bc,A	<0.001	2.21 bc,D	2.58 bc,C	2.94 bc,B	3.23 c,A	<0.001
T4	1.34 c,C	1.73 c,B	1.96 c,A	2.08 c,A	<0.001	1.94 c,C	2.11 c,C	2.77 c,B	3.07 cd,A	<0.001
T5	1.12 cd,D	1.54 cd,C	1.64 cd,B	1.82 cd,A	<0.001	1.66 cd,C	1.84 d,C	2.52 cd,B	2.88 d,A	<0.001
T6	0.85 d,C	1.29 d,B	1.48 d,A	1.53 d,A	<0.001	1.48 d,D	1.62 de,C	2.29 d,B	2.57 e,A	<0.001
*SEM*	0.21	0.23	0.59	0.40		0.42	0.60	0.07	0.10	
*p* value	<0.001	<0.001	<0.001	<0.001		<0.001	<0.001	<0.001	<0.001	

Means followed by different lower case in the same column indicate significant difference (*p* < 0.05), different uppercase letters in same raw within each parameter indicate significant differences (*p* < 0.05); T1: basal diet + 3.0 cm^3^ ZnNPs; T2: basal diet + 5.0 cm^3^ CurNPs; T3: basal diet + 2.0 cm^3^ Bl; T4: basal diet + 3.0 cm^3^ ZnNPs + 2 cm^3^ Bl; T5: basal diet +3.0 cm^3^ ZnNPs + 5.0 cm^3^ CurNPs and T6: basal diet +3.0 ZnNPs + 5.0 CurNPs + 2.0 cm^3^ Bl; TBC; Total bacterial count; TYMC: total yeast-mold count; *E. coli*: *Escherichia coli*; SEM: standard error mean. a–e, A–D: Different letters within one column are significantly different (*p* < 0.05). SEM: standard error means.

**Table 8:** Cecal microbiota (total bacteria, yeasts and molds, *E. coli*, coliform, *Salmonella* spp., *Enterococcus* spp., and lactic acid bacteria) are represented (Log CFU/g) broiler during feeding as affected by dietary supplementation of ZnNPs, CurNPs, and *Bacillus licheniformis*. (*n* = 5).

Samples	Total Bacteria	Total Yeasts and Molds	*E. coli*	*Salmonella* spp.	*Enterococcus* spp.	Coliform	Lactic Acid Bacteria
Control	9.20 a	3.92 a	5.70 a	1.58	5.82 a	6.70 a	4.51 d
T1	8.40 b	3.59 ab	5.50 ab	ND	5.60 b	6.51 ab	4.81 cd
T2	7.83 bc	3.52 b	5.30 b	ND	5.50 b	6.23 b	5.20 c
T3	7.65 c	3.41 bc	5.11 c	ND	5.21 bc	6.25 c	5.42 c
T4	7.40 cd	3.21 c	4.82 cd	ND	5.12 c	5.84 c	5.62 b
T5	7.25 d	3.00 cd	4.62 d	ND	4.90 c	5.55 cd	5.81 ab
T6	6.50 e	2.80 d	4.44 d	ND	4.71 d	5.26 d	6.18 a
SEM	0.17	0.22	0.14	0.20	0.30	0.19	0.20
*p* value	0.001	0.004	0.001	0.001	0.012	0.021	0.002

Basal diet (Control); T1: basal diet + 3.0 cm^3^ ZnNPs; T2: basal diet + 5.0 cm^3^ CurNPs; T3: basal diet + 2.0 cm^3^ Bl; T4: basal diet + 3.0 cm^3^ ZnNPs + 2 cm^3^ BL; T5: basal diet +3.0 cm^3^ ZnNPs + 5.0 cm^3^ CurNPs and T6: basal diet + 3.0 ZnNPs + 5.0 CurNPs + 2.0 cm^3^ Bl; *E. coli*: *Escherichia coli*; SEM: standard error mean. a–e: Different letters within one column are significantly different (*p* < 0.05).

## Data Availability

Not applicable.
